# Supplementary material for: Repurposing of the Cardiovascular Drug Statin for the Treatment of Cancers: Efficacy of Statin–Dipyridamole Combination Treatment in Melanoma Cell Lines
Source: Biomedicines. 2024 Mar 21;12(3):698. doi: 10.3390/biomedicines12030698 (PMC10968169; doi:10.3390/biomedicines12030698)
Supplement: Supplementary file 1 [file biomedicines-12-00698-s001.zip › biomedicines-2909336-supplementary.pdf]

biomedicines

MDPI

Article

Supplementary Material: Repurposing of the Cardiovascular Drug Statin for the Treatment of Cancers: Efficacy of Statin–Dipyridamole Combination Treatment in Melanoma Cell Lines

Nanami Irie, Kana Mizoguchi, Tomoko Warita, Mirai Nakano, Kasuga Sasaki, Jiro Tashiro, Tomohiro Osaki, Takuro Ishikawa, Zoltán N. Oltvai, and Katsuhiko Warita

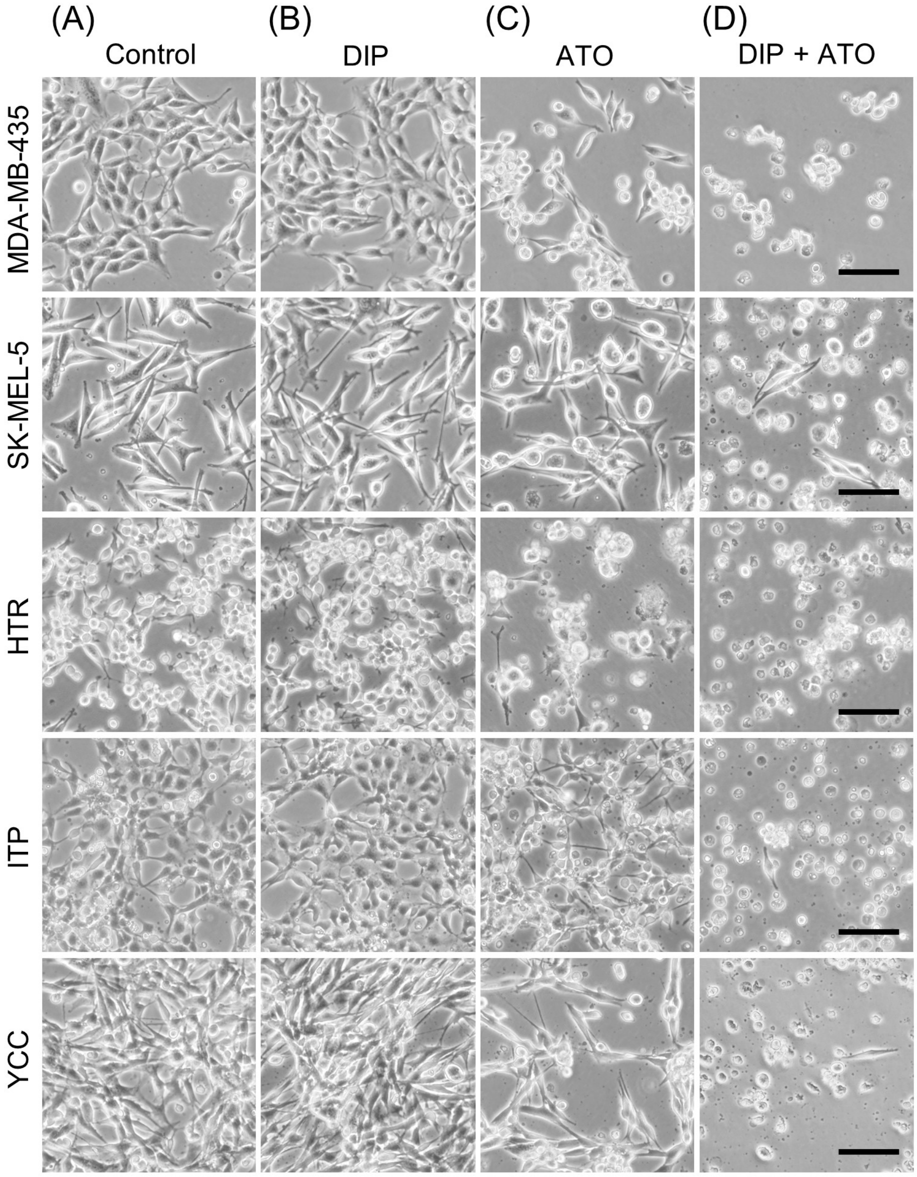

Figure S1. Changes in the morphology of dipyridamole-, atorvastatin-, and dipyridamole-and-atorvastatin-treated cells. Morphologies of human melanoma cells (MDA-MB-435 and SK-MEL-5) and canine melanoma cells (HTR, ITP, and YCC) are shown after a 72 h of 3 μM dipyridamole [DIP]- (B), 10 μM atorvastatin [ATO]- (C), or DIP+ATO co-treatment (D). Control cells were treated with 0.2% DMSO (A). Images of the cell morphology were taken in the field of 80% confluence. Scale bar = 100 μm.

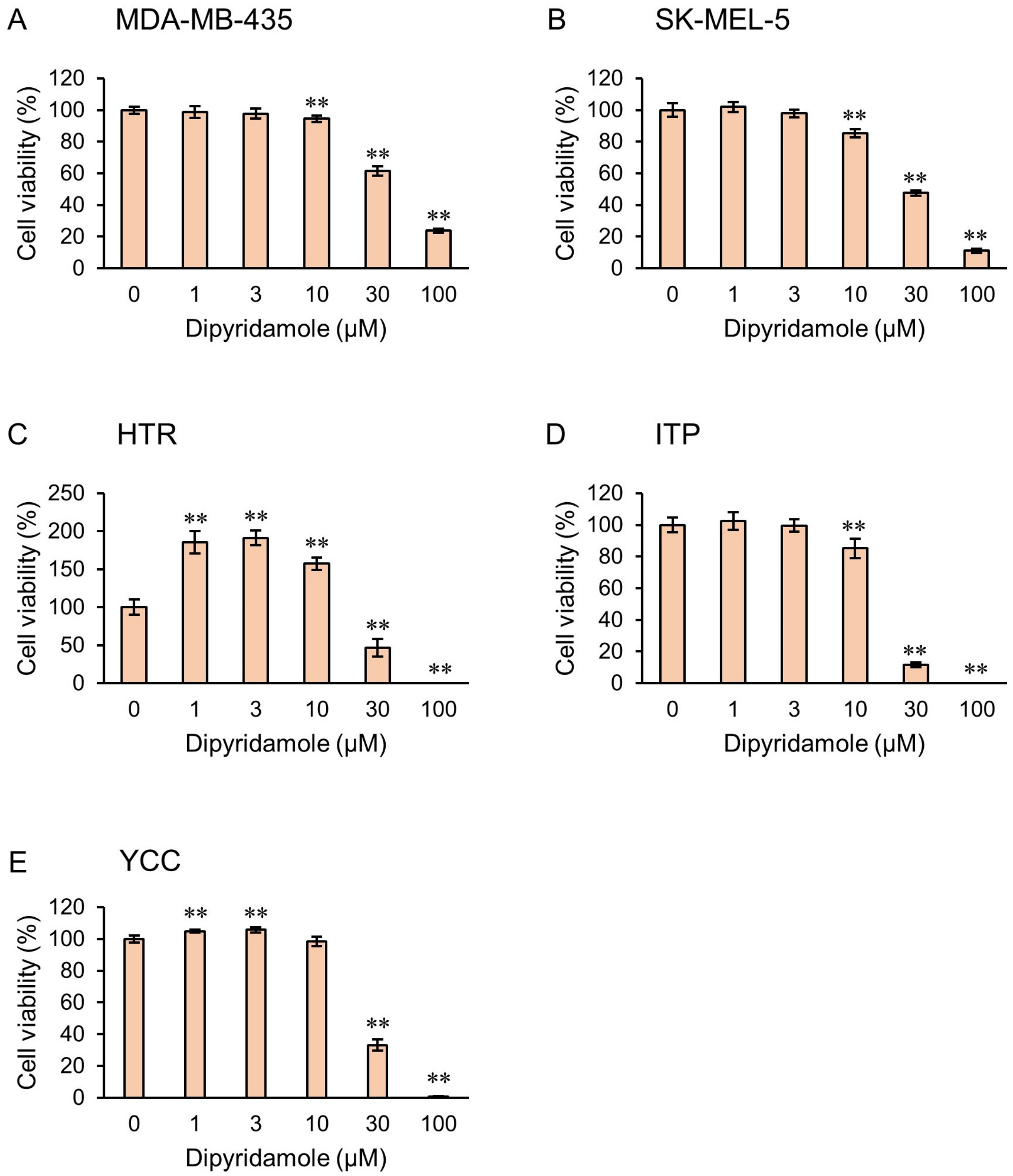

Figure S2. Effect of dipyridamole treatment on growth of human and canine melanoma cells. The relative growth of human (MDA-MB-435 (A) and SK-MEL-5 (B)) and canine (HTR (C), ITP (D), and YCC (E)) melanoma cell lines is shown. All cell lines were treated with 1–100 µM dipyridamole for 72 h. Values in DMSO control were set to 100%. Each value represents the mean ± SD (n = 3). Data were analyzed using two-way analysis of variance (ANOVA) with Dunnett's test for multiple group comparisons. **$P < 0.01$, with respect to a control group.

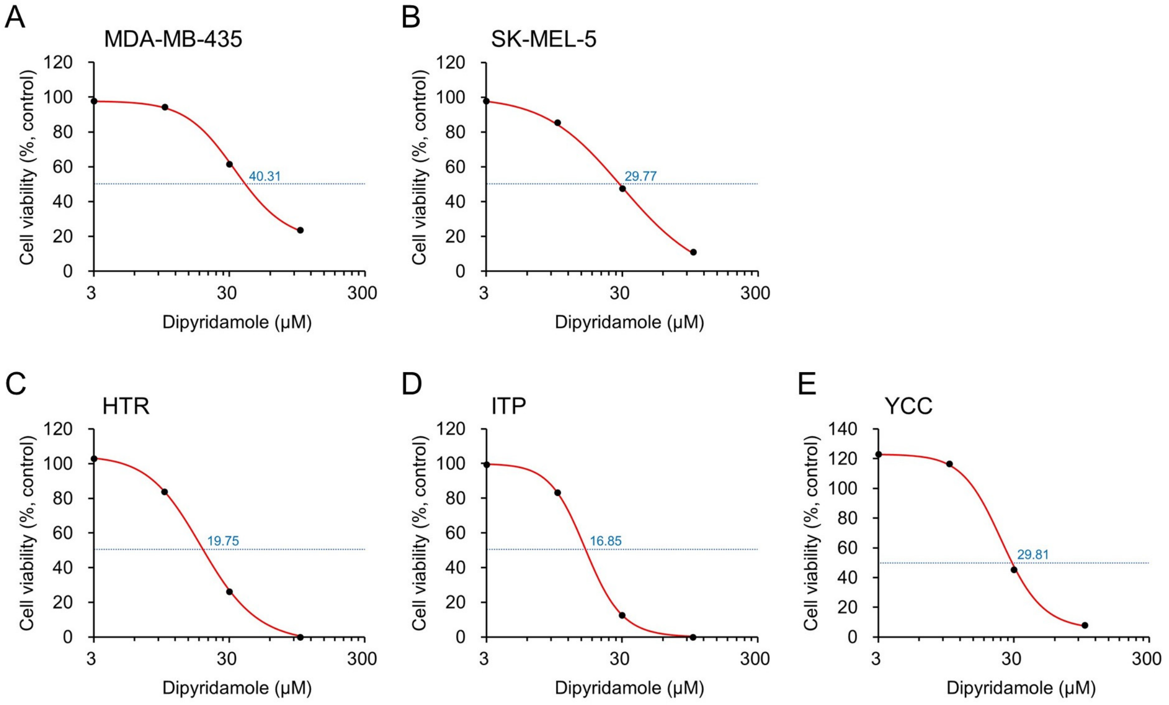

Figure S3. Determination of half maximal inhibitory concentration (IC_{50}) value of dipyridamole on human (A and B) and canine (C, D, and E) melanoma cells. After treatment with dipyridamole on cells for 72 h, the percentage of cell viability was determined by CCK-8 assay using WST-8. The IC_{50} value of dipyridamole was determined by fitting a sigmoidal curve to the data using the ImageJ software.

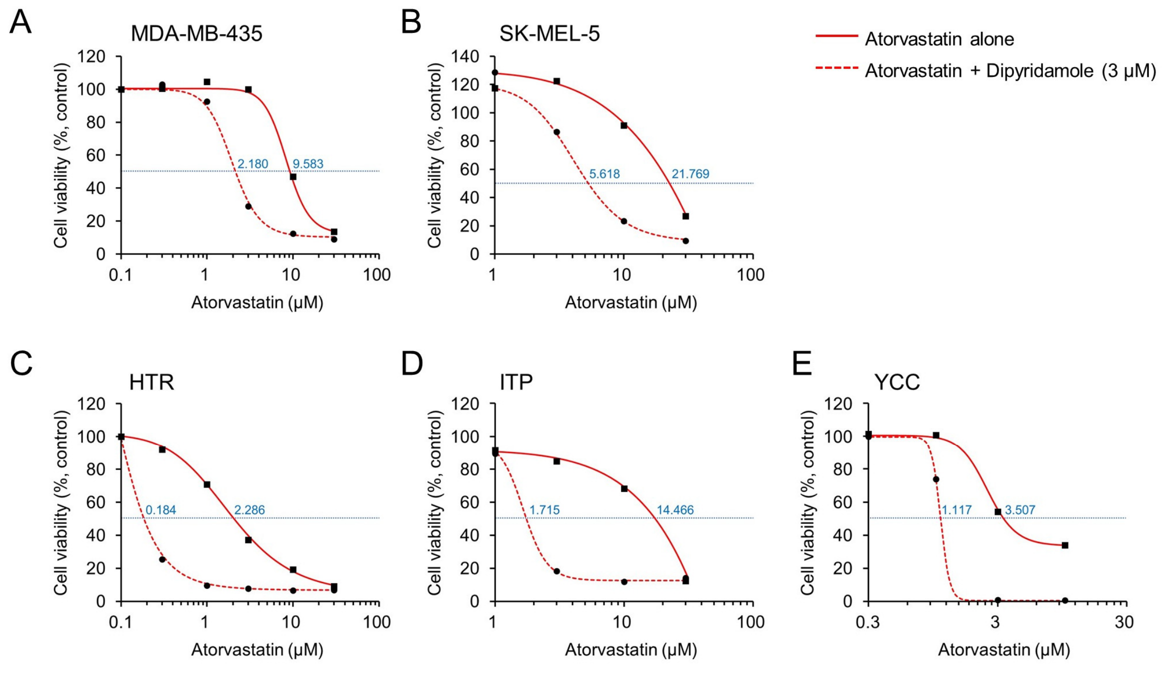

Figure S4. Determination of IC_{50} value of atorvastatin in atorvastatin single treatment or combined treatment with dipyridamole in human (A and B) and canine (C, D, and E) melanoma cells. After treatment with 0.3–30 μM atorvastatin with or without 3 μM dipyridamole on cells for 72 h, the percentage of cell viability was determined by CCK-8 assay using WST-8. The IC_{50} value of atorvastatin was determined by fitting a sigmoidal curve to the data using the ImageJ software. The solid line represents atorvastatin treatment and the dotted line represents atorvastatin plus dipyridamole treatment.

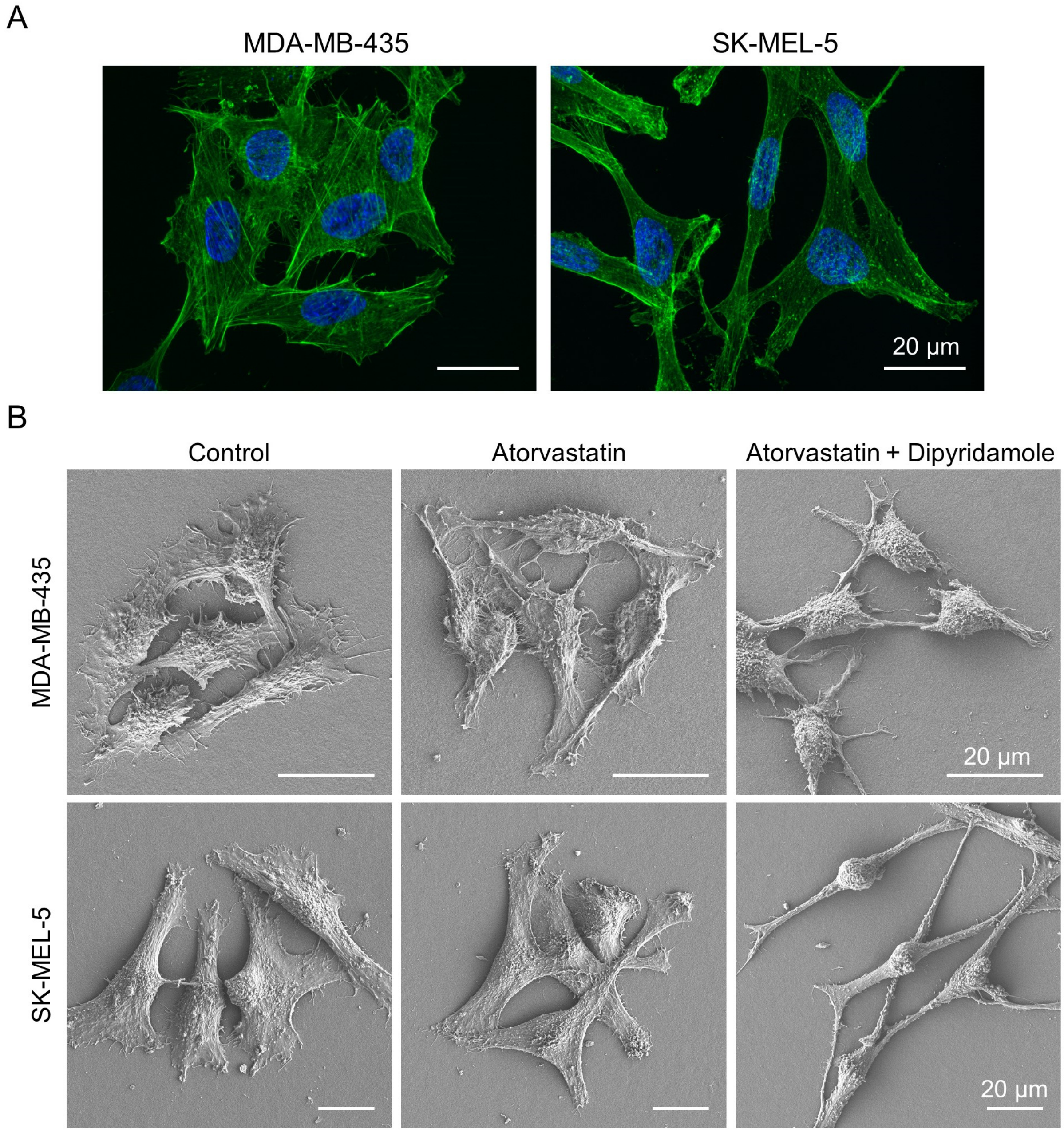

Figure S5. Images of actin filaments, filopodia, and lamellipodia in human melanoma cells. Actin filaments of MDA-MB-435 and SK-MEL-5 cells stained by falloidin (A) and filopodia and lamellipodia of MDA-MB-435 and SK-MEL-5 cells after 5 μM atorvastatin and 5 μM atorvastatin plus 3 μM dipyridamole treatment for 24 h, as detected by SEM (B) are shown. The actin filaments of MDA-MB-435 cells were more numerous and clearer than those of SK-MEL-5 cells (A). In MDA-MB-435 and SK-MEL-5 cells treated with atorvastatin alone, filopodia and lamellipodia were clearly affected, and in those treated with a combination of atorvastatin and dipyridamole, the morphology changed considerably, and filopodia and lamellipodia were not clearly observed (B, middle and right panels). Control cells were treated with 0.2% DMSO (B, left panels).

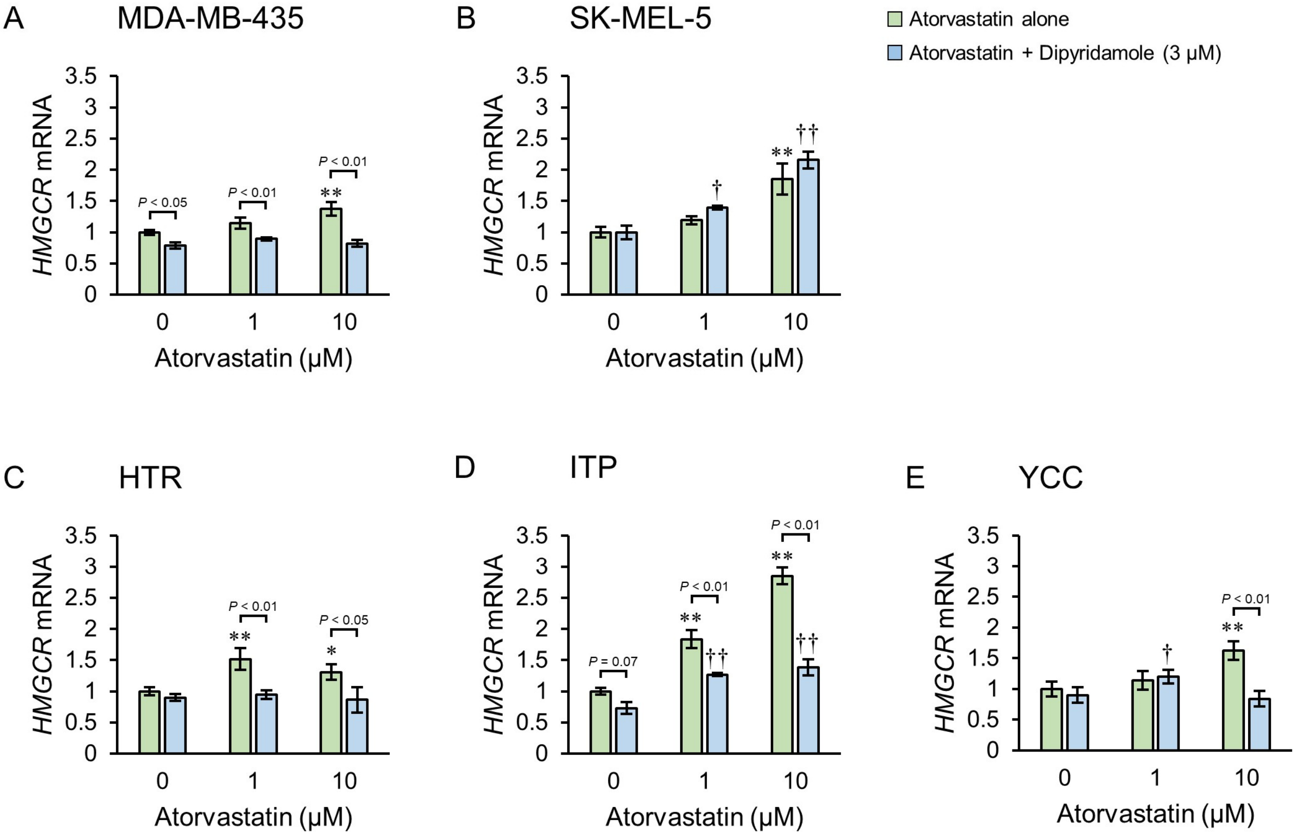

Figure S6. Effect of atorvastatin and/or dipyridamole on *HMGCR* mRNA expression in human and canine melanoma cells. *HMGCR* levels in the indicated human (A and B) and canine (C, D, and E) melanoma cells treated with atorvastatin alone or in combination with dipyridamole for 24 h, were normalized by *RPLP2* levels for human cells and *GAPDH* levels for canine cells. The measured values of the experimental group were compared with those of the control group using a one-way analysis of variance (ANOVA) with the Tukey–Kramer post hoc test for multiple group comparisons. Data are presented as the mean ± SD (n = 3). $^{*}P < 0.05$, $^{**}P < 0.01$, $^{\dagger}P < 0.05$, $^{\dagger\dagger}P < 0.01$, with respect to each control group.
